# Drug resistance to paclitaxel is not only associated with *ABCB1* mRNA expression but also with drug accumulation in intracellular compartments in human lung cancer cell lines

**DOI:** 10.3892/ijo.2011.1297

**Published:** 2011-12-13

**Authors:** MASANORI SHIMOMURA, TAKESHI YAOI, KYOKO ITOH, DAISHIRO KATO, KUNIHIKO TERAUCHI, JUNICHI SHIMADA, SHINJI FUSHIKI

**Affiliations:** 1 Department of General Thoracic Surgery, Graduate School of Medical Science, Kyoto Prefectural University of Medicine, 465 Kajii-cho, Kawaramachi-Hirokoji, Kamigyo-ku, Kyoto 602-8566, Japan; 2 Department of Pathology and Applied Neurobiology, Graduate School of Medical Science, Kyoto Prefectural University of Medicine, 465 Kajii-cho, Kawaramachi-Hirokoji, Kamigyo-ku, Kyoto 602-8566, Japan

**Keywords:** paclitaxel, drug resistance, intracellular localization, lung cancer

## Abstract

In order to clarify the mechanisms of resistance to paclitaxel in lung cancer, three human lung cancer cell lines which exhibit different sensitivity to paclitaxel were investigated from the following viewpoints: overexpression of ATP-binding cassette, sub-family B, member 1 (*ABCB1*), mutations on paclitaxel binding site of β-tubulin genes, quantity of polymerized tubulin and the intracellular localization of paclitaxel. *ABCB1* expression was evaluated by real-time RT-PCR. No correlations were noted between the *ABCB1* expression in the sensitive and resistant cell lines at the mRNA level. No mutations on the paclitaxel binding site of the β-tubulin genes were detected in either the resistant or sensitive cells. Live cell images obtained by confocal laser microscopy revealed that the resistant cell line, RERF-LC-KJ, had more accumulation of Oregon Green^®^ 488 conjugated paclitaxel in the lysosomal and extra-lysosomal compartments of cytoplasm than other cell lines. The results obtained in this study indicated that the changes in the subcellular localization could contribute to the production of paclitaxel resistance in lung cancer cell lines. Further studies should be conducted to elucidate the molecular mechanisms that differentiate the intracellular localization of paclitaxel.

## Introduction

Lung cancer is one of the most aggressive cancers and the world’s leading cause of cancer mortality. Nevertheless, the treatment of lung cancer still remains unsatisfactory. The 5-year survival rate for lung cancer is reported to be less than 50% in Japan (1). Paclitaxel is a potent anti-cancer agent that binds to β-tubulin and prevents mitosis through microtubule over-stabilization. Paclitaxel is known to be effective as a cancer chemotherapeutic agent for ovarian, breast, gastric and lung cancers, but paclitaxel-based chemotherapy for lung cancer is not as effective as it is for other cancer types, with a response rate of 30–40% (2), since tumor cells develop mechanisms of resistance to the agent.

Several mechanisms have been suggested for this resistance to paclitaxel. The first mechanism reported as a mediator of the resistance to paclitaxel was the overexpression of the multidrug transporter gene, encoding for an efflux pump able to efflux paclitaxel, thereby hampering drug retention. Studies have shown that both the multidrug transporter ATP Binding Cassette, sub-family B, member 1 (*ABCB1*, *MDR1*) (3,4) and the ATP Binding Cassette, sub-family C, member 1 (*ABCC1*, *MRP1*) (5) play a role in enhancing the cellular efflux of anti-cancer drugs, including paclitaxel. The second mechanism proposed indicated point mutation in the β-tubulin gene at the paclitaxel binding site as being responsible for the resistance to paclitaxel (6). A correlation was demonstrated between β-tubulin point mutation and resistance to paclitaxel in lung cancer patients (7). *In vitro* studies on human cell lines demonstrated that the resistance to paclitaxel resulted from mutations in human class I (M40) β-tubulin, the predominant isotype, and class IVa β-tubulin, thereby causing changes in the microtubule dynamics and stability (8–15). Furthermore, altered expression levels of tubulin isotypes have been associated with the development of the resistance to paclitaxel (6,16). Therefore, we addressed the issue of the mechanism of the resistance to paclitaxel by employing three human non-small cell lung cancer cell lines, each of which exhibited a different sensitivity to paclitaxel.

First, we studied the expression of drug transporters and β-tubulin mutations in the cell lines. However, no correlation was shown between the expression of *ABCB1* or *ABCC1* and the resistance to paclitaxel, and sequencing of β-tubulin failed to disclose any mutation in the paclitaxel binding site in any of these cell lines. Therefore, we further investigated the intracellular pharmacokinetics of paclitaxel by measuring the intracellular accumulation of paclitaxel and stabilized tubulin, and by observing live cells treated with fluorescence-labeled paclitaxel. We thus obtained the novel finding that fluorescence-labeled paclitaxel was accumulated more in the lysosomal and extra-lysosomal compartments in cells showing a resistance to paclitaxel, compared with other cells.

## Materials and methods

### Cell lines and cell cultures

Human lung cancer cell lines II18, PC-14 and RERF-LC-KJ were purchased from RIKEN Cell Bank (Tsukuba, Japan) and RERF-LC-Ad1, A549, RERF-LC-Ad2 and Lu99B were purchased from the Health Science Research Resources Bank (Osaka, Japan). A549 was cultured in Eagle’s minimal essential medium (MEM) supplemented with 10% fetal bovine serum (FBS). The other cell lines were cultured in RPMI-1640 with 10% FBS. All of the media were supplemented with penicillin-streptomycin sulfate (Nacalai Tesque Inc., Kyoto, Japan). All of the cell lines were incubated in an atmosphere of 95% air with 5% CO_2_ at 37°C.

### Cytotoxicity assay and real-time monitoring of cell proliferation to paclitaxel

Paclitaxel was obtained from Bristol-Myers Squibb (Tokyo, Japan). Drug cytotoxicity for paclitaxel was measured using 2-(2-methoxy-4-nitrophenyl)-3-(4-nitrophenyl)-5-(2,4-disulfophenyl)-2H-tetrazolium (WST-8) assays (Dojindo Laboratories, Kumamoto, Japan), according to the manufa-cturer’s protocol. Cells were harvested with trypsin and re-suspended at a final concentration of 2.5×10^4^ cells/ml for A549 and RERF-LC-KJ, a final concentration of 5.0×10^4^ cells/ml for RERF-LC-Ad1, RERF-LC-Ad2 and Lu99B, and a final concentration of 1.0×10^5^ cells/ml for II18 and PC-14, in order to assure appropriate absorbance ranging from 1.0 to 2.0 at 450 nm. Aliquots of each of the suspended cells (100 μl) were prepared in triplicate and distributed into 96-well microplates. After incubation for 24 h, the cells were exposed to paclitaxel at various concentrations (ranging from 0.1 nM to 1 mM, 8 different concentrations) for 48 h. Subsequently, we then refreshed the medium, followed by adding 10 μl WST-8 to each well and incubation for 2 h at 37°C. The absorbance at 450 nm was determined by a microplate reader (Bio-Rad Laboratories, Tokyo, Japan). The concentrations required to inhibit growth by 50% (IC_50_) were calculated using KaleidaGraph version 4.0 (Synergy Software, Reading, PA).

The xCELLigence^®^ system (Roche Diagnostics, Tokyo, Japan) was used to quantitatively measure the cell proliferation. Cells (8×10^4^-1.3×10^3^ cells per well) were prepared in triplicate and distributed into E-plate 96 microplates (Roche Diagnostics) to measure the cell index (reflecting the surface area covered by the cells) in each well after 96 h of incubation. In the cytotoxicity assay, cells were re-suspended at 1×10^4^ cells for PC-14, A549 and RERF-LC-Ad2, 2×10^4^ cells for II18 and Lu99B and 4×10^4^ cells for RERF-LC-Ad1 and RERF-LC-KJ, respectively, in order to assure an appropriate cell index. Cells were prepared in triplicate, with 200 μl of an appropriate medium with 10% FBS. After 24 h of incubation, the cells were exposed to 25 μl of paclitaxel at various concentrations. The cells were monitored every 15 min (up to 48 h), followed by monitoring at 1-h intervals (from 48 to 96 h). IC_50_ was calculated using the RTCA-integrated software of the xCELLigence^®^ system.

### Total RNA preparation from paclitaxel-exposed cells

We harvested the cells exposed to 3.2 nM (−8.5 logM) paclitaxel at different time points, at 0, 6, 12, 18, 24 and 48 h in culture, respectively. The total cellular RNA was isolated from the cells using the RNeasy Mini Kit (Qiagen, Tokyo, Japan). After treatment with RNase-free DNase I (Nippongene, Tokyo, Japan) for 30 min at 37°C, the RNA was processed for purification with an RNeasy MinElute Cleanup Kit (Qiagen).

### Real-time quantitative RT-PCR

First-strand cDNAs were synthesized with a Superscript VILO^®^ cDNA synthesis kit (Invitrogen, Tokyo, Japan). Real-time quantitative PCR of the target genes was performed with an ABI PRISM 7000 (Applied Biosystems, Carlsbad, CA) and a SYBR Premix Ex Taq Perfect Real-Time (Takara-Bio, Otsu, Japan). PCR reactions were carried out under the following conditions: 95°C for 10 sec, followed by 40 cycles of 95°C for 5 sec and 65°C for 35 sec. The PCR primers were designed using Primer 3 Plus software^®^ as follows: *ABCB1*, 5′-TCCTGGAGCGGTTCTACGAC-3′ (sense) and 5′-GCTGCAGTCAAACAGGATGG-3′ (antisense); *ABCC1*, 5′-GGAGACCTGGAAGCTGATGG-3′ (sense) and 5′-AGGGCTCCATAGACGCTCAG-3′ (antisense); *GAPDH*, 5′-GAAGGTGAAGGTCGGAGTC-3′ (sense), 5′-GAAGAT GGTGATGGGATTTC-3′ (antisense). The results were normalized to the levels of *GAPDH* and relative quantification was calculated using the ΔΔCT method (17). Relative mRNA expressions were expressed as fold changes relative to the expression level of the gene on A549 at time zero.

### Sequencing of β-tubulin

The primers employed for the PCR amplification and sequencing of the β-tubulin isotype are summarized in Table I. The total RNA (1 μg) was reversely transcribed to cDNA using a SuperScript^®^ II RNase H-Reverse Transcriptase Kit (Invitrogen). PCR products were synthesized using KOD-plus (Toyobo, Osaka, Japan) according to the manufacturer’s protocols, and loaded onto 2% agarose gel. Following electrophoresis, the appropriate bands were purified using Wizard^®^ SV Gel and PCR Clean-Up System (Promega, Madison, WI) according to the manufacturer’s instructions. The products were purified by ethanol precipitation and added to BigDye^®^ Terminator v3.1 Cycle Sequencing Kit (Applied Biosystems) and then processed for sequencing using an Applied Biosystems 3130 Genetic Analyzer (Applied Biosystems). Sequences of the β-tubulin isotype were aligned using Clustal-W2 (European Bioinformatics Institute) and Boxshade programs.

### Paclitaxel accumulation assay

The accumulation of paclitaxel in all of the cells was measured using [^3^H]-paclitaxel (Moravec Biochemicals Inc., Brea, CA), employing a modification of the methods previously described (18,19).

First, the cells were quadricated and distributed into 96-well plates at the described cellular count for a cytotoxic assay with xCELLigence^®^ system. After incubation for 24 h, the cells were exposed to 3.2 nM (−8.5 logM) [^3^H]-paclitaxel for the following 6, 12, 18, 24 and 48 h. After washing three times with ice-cold phosphate-buffered saline (PBS), the cells were trypsinized, and then triplicated and solubilized with 1% Triton-X and 0.2% sodium dodecylsulfate (SDS) in 10 mM PBS (pH 7.4), except for one well, which was employed for cell counting. Each solution was transferred to Optiplate-96^®^ (Perkin-Elmer, Waltham, MA) and added to Microscinti 40^®^ (Perkin-Elmer). The radioactivity was measured as counts per min (CPM) using a TopCount NXT^®^ (Perkin-Elmer, Inc.) microplate scintillation and luminescence counter.

### Tubulin polymerization assay

Soluble and polymerized tubulins from cell lysates were divided by the following procedures based on a modification of the previously reported method (6,13,20). In brief, cells (1×10^6^ cells) exposed to 3.2 nM (−8.5 logM) paclitaxel for 0, 6, 12, 18, 24 and 48 h were lysed at 37°C with 100 μl of hypotonic buffer [1 mM MgCl_2_, 2 mM EGTA, 0.5% NP-40, a complete mini protease inhibitor cocktail tablet (Roche Diagnostics) and 20 mM Tris-HCl (pH 6.8)] for 5 min in the dark. After an additional 100 μl of hypotonic buffer was added, the cell lysates were vortexed briefly and sonicated on ice. Following quantification of the protein concentration, the lysate was centrifuged at 14,000 rpm for 10 min at room temperature. We collected proteins from the supernatant, which contained the soluble tubulin, and the pellet fraction, including polymerized tubulin. Proteins were solubilized in 4X NuPAGE^®^ LDS Sample Buffer (Invitrogen) and 10X NuPAGE^®^ Reducing Agent (Invitrogen) and then sonicated on ice. Heat-denatured proteins (10 μl) were separated using the NuPAGE^®^ system and NuPAGE^®^ Novex 4–12% Bis-Tris Gel (Invitrogen), and then transferred onto Hybond-P (GE Healthcare Ltd., Buckinghamshire, UK). SNAP-id (Millipore, Billerica, MA) was used for immunoblotting. A mouse monoclonal anti-α-tubulin antibody (1:500) (Sigma-Aldrich, St. Louis, MO) was applied and incubated for 10 min. After the secondary reaction with ECL^®^ peroxidase-labelled anti-mouse NA931VS (1:1000) (GE Healthcare Ltd.) for 10 min, the chemiluminescent signal was visualized by using SuperSignal West pico^®^ (Thermo Fisher Scientific Inc., Waltham, MA) on Versadoc (Bio-Rad Laboratories). We analyzed the protein expression using Quantity One software (Bio-Rad Laboratories).

### Measurement of acetylated tubulin

The amount of acetylated tubulin was measured by the modified procedure previously described (14). Proteins were extracted from the total cell lysate using a ProteoExtract^®^ Protein Precipitation Kit (Merck KGaA, Darmstadt, Germany). After transferring the proteins to Hybond-P, the blots were incubated with mouse monoclonal anti-acetylated α-tubulin antibody (1:1000) (Abcam, Cambridge, UK) and anti-α-tubulin antibody (Sigma-Aldrich), which was used for the tubulin polymerization assay. The secondary reactions and the signal detection were performed similarly to the method described in the section on the tubulin polymerization assay.

### Imaging of acetylated tubulin on immunofluorescence microscopy

Seeded on glass coverslips, the cells were exposed to paclitaxel for 6 and 24 h. After the exposure, the cells were fixed with 4% paraformaldehyde and stained with mouse monoclonal anti-acetylated α-tubulin antibody (1:1000). Alexa 488 conjugated goat anti-mouse IgG was used as a secondary antibody. In order to stain α-tubulin with the antibody described above, we used Zenon^®^ Mouse IgG Labeling Kits (Molecular probes, Eugene, OR) according to the manufacturer’s manual. In order to visualize the nuclei, the cells were stained with 4′,6-diamidino-2-phenylindole (DAPI). The immunostained cells were observed and analyzed with a confocal laser microscope LSM 510 (Carl-Zeiss AG, Oberkochen, Germany) and LSM image browser software.

### Live cell imaging under confocal laser microscope

All of the cell lines were grown to confluency in culture dishes and trypsinized. Then, 1×10^4^ cells were distributed into 4-well glass-bottom dishes (Matsunami Glass Ind., Inc., Osaka, Japan) coated with poly-L-lysine. After incubation for 24 h, each well was changed to pre-warmed fresh medium containing 100 nM Oregon Green^®^ 488 conjugated paclitaxel (Tubulin Tracker™) (Molecular probes) and incubated for 6 and 24 h, with the addition of 100 nM LysoTracker^®^ Red DND-99 (Molecular probes) for 2 h. After the incubation, DAPI was added to the media, followed by incubation for another 30 min. Each dish was washed three times with PBS and observed immediately under the confocal laser microscope LSM 510.

### Statistical analysis

The correlation coefficient between the IC_50_ value shown by the WST-8 assay and xCELLigence^®^ system was obtained using simple regression analysis (Excel software). In order to determine if the differences in the intracellular accumulation of paclitaxel and gene expressions among the cell lines were statistically significant, the data was analyzed employing one-way analysis of variance, followed by Dunnett’s multiple comparisons test.

## Results

### Remarkably different resistance to paclitaxel between lung cancer cell lines

In order to study the mechanisms underlying paclitaxel-induced cytotoxicity, we first compared the cellular responses to paclitaxel with two independent assays. Seven lung cancer cell lines from human non-small lung cancers were studied to assess the IC_50_, and it turned out that their IC_50_ were widely distributed in the range from −3.70 to −8.33 logM and −4.48 to −10.80 logM, according to the xCELLigence^®^ system and the WST-8 assay, respectively (Table II). Simple regression analysis showed a strong positive correlation (r^2^=0.771) in both cytotoxicity assays.

**Figure 1:**
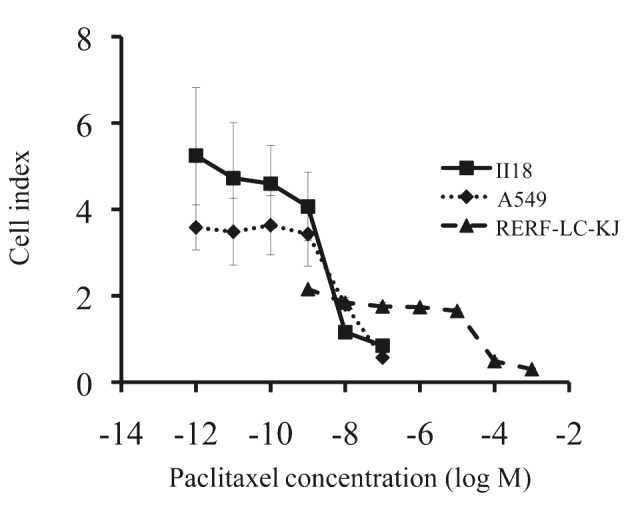
The effect of paclitaxel varied remarkably between the three lung cancer cell lines. The results of xCELLigence^®^ system were used to measure the cell index, defined as the value reflecting the surface area covered by the living cells. Points, mean of one triplicated experiment; bars, SD.

**Figure 2:**
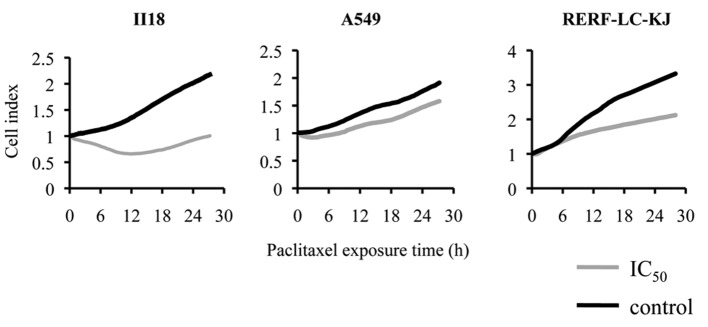
The cytotoxicity of paclitaxel under concentrations required to inhibit growth by 50% (IC_50_) appeared most prominently within 24 h after the exposure, regardless of the cell line. The cellular proliferation was monitored by xCELLigence^®^ system. After 24 h of incubation, the cells were exposed to paclitaxel.

RERF-LC-KJ, the most resistant cell line among the cell lines so far examined, was about 10^4^ times more resistant to paclitaxel than II18, the most sensitive cell line (Fig. 1). After examining the effect of paclitaxel at calculated IC_50_ on cellular proliferation with the xCELLigence^®^ system, we concluded that the cytotoxicity appeared most prominently between 6 and 18 h after the exposure to paclitaxel, regardless of the cell line (Fig. 2). Thus, we assumed that the most remarkable changes in cellular behavior appeared in the period before 24 h. Because a similar tendency in regard to the resistance to paclitaxel was observed with two different assays, we concluded that there was a wide variation in terms of the sensitivity (or resistance) to paclitaxel among the different lung cancer cell lines. We then decided to use three cell lines, i.e., RERF-LC-KJ, A549, and II18 for the following experiments. Taking into consideration the IC_50_ of the most sensitive cell line II18, we determined the concentration of paclitaxel being as 3.2 nM (−8.5 logM).

### ABCB1 expression level change is not related to resistance to paclitaxel

**Figure 3:**
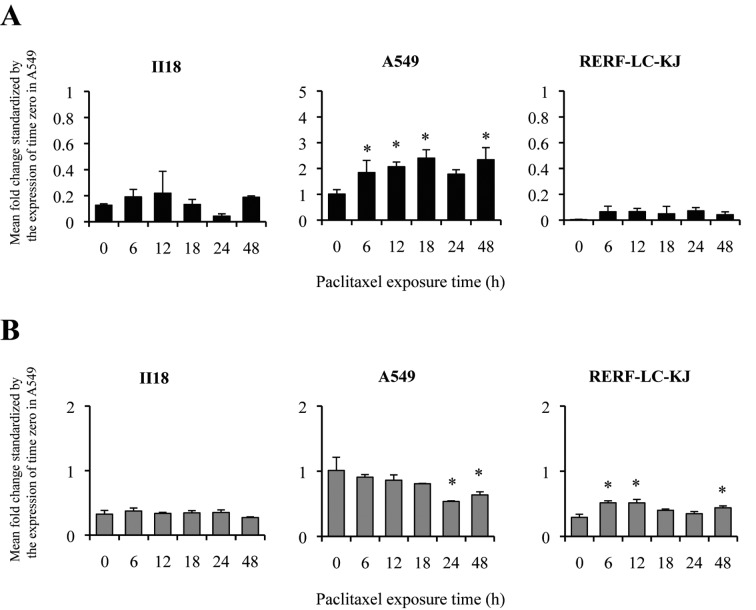
The mRNA levels of *ABCB1* (A) changed with time and differed in each lung cancer cell line when exposed to 3.2 nM paclitaxel. The changes in the mRNA level of *ABCC1* (B) were modest in the II18 and RERF-LC-KJ cell lines, whereas the expression level significantly decreased at 24 and 48 h in the A549 cell line. The mRNA levels of *ABCB1* and *ABCC1* were analyzed by real-time RT-PCR and calculated by the ΔΔCT method. The data are presented relative to the level of *ABCB1* and *ABCC1* at time zero in the A549 cell line. The expression levels were normalized by the housekeeping gene *GAPDH*. Columns, the mean of one triplicated experiment; bars, SD; ^*^p<0.05 relative to respective untreated (0 h) cells.

To further elucidate the relationship between the major drug transporters and the resistance to paclitaxel, we measured the expression of *ABCB1* (Fig. 3A) and *ABCC1* (Fig. 3B) in lung cancer cell lines by quantitative real-time RT-PCR. A549 showed an elevation of the expression level of *ABCB1* between 6 and 12 h. On the other hand, II18 and RERF-LC-KJ remained low throughout the experimental period in terms of the expression level of *ABCB1*.

However, no correlation was shown between the patterns of the expression level of *ABCB1* and the resistance to paclitaxel. The expression level of *ABCC1* at time 0 for A549 was significantly higher than that of the other cell lines. The ratio of the expression level at time 0 for *ABCC1* compared to A549 remained in the range from 0.18 to 0.58, and the changes in the expression level were modest.

### Accumulation of [^3^H]-paclitaxel corresponded with the expression of ABCB1 in two cell lines

**Figure 4:**
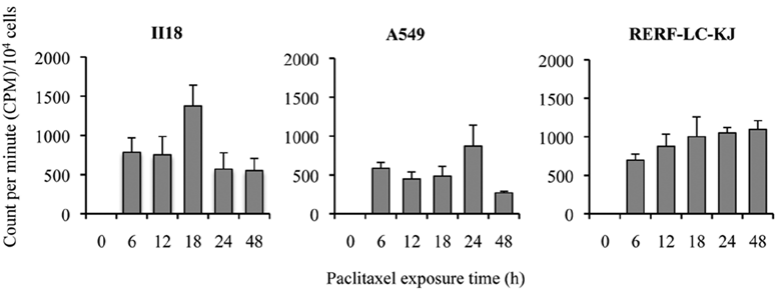
The intracellular accumulation of 3.2 nM [^3^H]-paclitaxel increases with time in the RERF-LC-KJ cell line, until it reached a peak between 12–24 h. The values were calculated as the CPM value divided by the cell number of each well. Columns, the mean of one triplicated experiment; bars, SD.

In order to investigate whether the accumulation of paclitaxel was regulated by *ABCB1* and *ABCC1*, and whether the degree of accumulation was correlated to the development of the resistance to paclitaxel, we quantitatively analyzed the time course of the accumulation of intracellular [^3^H]-paclitaxel. The accumulation was evaluated as the radioactivity of [^3^H]-paclitaxel using CPM divided by the cell count for each well. The accumulation of [^3^H]-paclitaxel increased gradually and continuously with time for RERF-LC-KJ, whereas it increased until it reached a peak between 12 or 24 h and then decreased in the other cell lines (Fig. 4). RERF-LC-KJ showed a low expression of *ABCB1* throughout the entire time course of the experiment, which corresponded with a higher accumulation of [^3^H]-paclitaxel. The correlation coefficient between the expression level of *ABCB1* and the accumulation of [^3^H]-paclitaxel was −0.804. On the contrary, the accumulation of [^3^H]-paclitaxel in A549 remained low, which correlated well with the high expression level of *ABCB1*. However, this inverse relationship between the accumulation of [^3^H]-paclitaxel and the expression of *ABCB1* did not apply to the II18 cell line. The correlation coefficient between the expression level of *ABCC1* and the accumulation of [^3^H]-paclitaxel was −0.526. Thus, we concluded that there was a stronger correlation between the expression of *ABCB1* and the intracellular accumulation of paclitaxel compared to that between the expression of *ABCC1* and the intracellular accumulation of paclitaxel.

### Changes in the quantity of polymerized tubulin and acetylated tubulin after exposure to paclitaxel are not related to the resistance to paclitaxel

**Figure 5:**
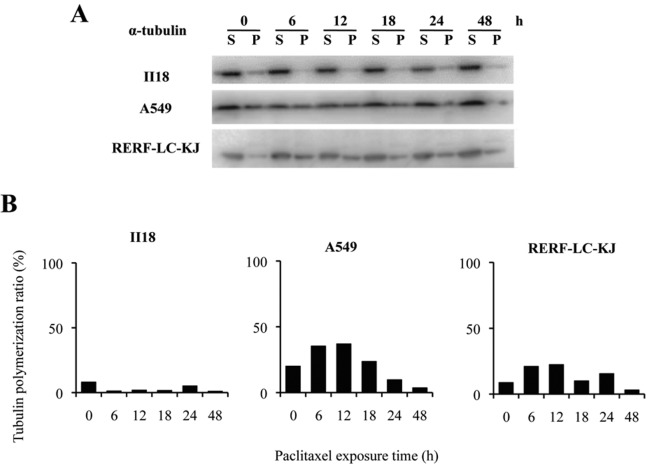
Paclitaxel does not stabilize the microtubules in the II18 cell line for 48 h, whereas it does in the other cell lines, with a peak between 6 or 12 h. (A), Immunoblots of proteins from lung cancer cell lines with antibodies against α-tubulin. The loading quantity was equal to 10 μg proteins from three 3.3 μg total proteins from the cell lines exposed to 3.2 nM paclitaxel. Cells were lysed in hypotonic buffer for 5 min at 37°C followed by separation of the soluble (S) and polymerized (P) fractions by centrifugation. (B), The percentage of polymerized tubulin was obtained by dividing the densitometric value of the polymerized tubulin (insoluble) by the total tubulin contents.

In the II18 cell line, no correlation was found between the expression level of *ABCB1* and the extent of the accumulation of [^3^H]-paclitaxel. Therefore, we next performed tubulin polymerization assays to quantify the extent of microtubule stabilization by paclitaxel. The tubulin-polymerized ratio was defined as the percentage of polymerized α-tubulin, which was calculated by dividing the densitometric value of polymerized α-tubulin (insoluble) by the total α-tubulin contents (sum of the densitometric values for soluble and polymerized α-tubulin) (Fig. 5). The ratio of polymerized tubulin remained low for 48 h in the II18 cell line, whereas it increased until a peak value at 6 or 12 h after the exposure and decreased from 18 to 48 h in the other two cell lines.

**Figure 6:**
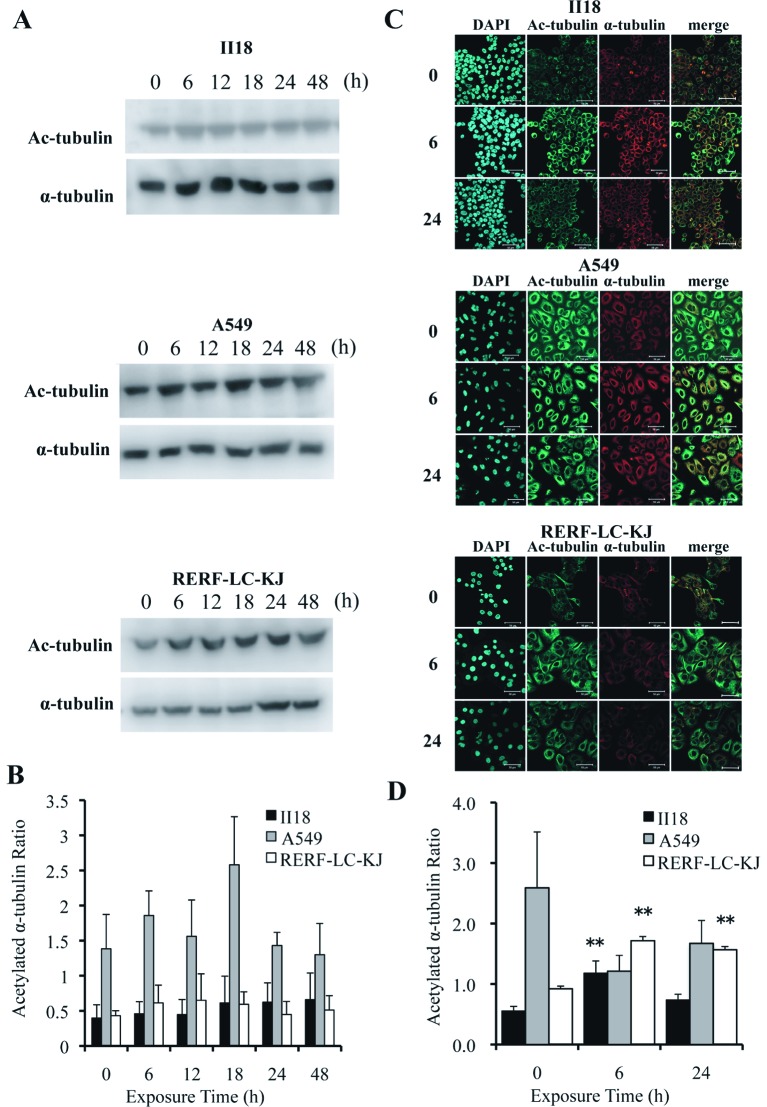
The expression of acetylated α-tubulin remained low for 48 h in the II18 cell line, whereas it increased with time in the RERF-LC-KJ cells. (A), The expressions of acetylated α-tubulin and α-tubulin were analyzed with the Western blot method using proteins extracted from cells exposed to 3.2 nM (−8.5 logM) paclitaxel for 6, 12, 18, 24 and 48 h and untreated (0 h). (B), The ratio of the expression level of acetylated α-tubulin to the total α-tubulin was measured. Columns, the mean of value of three experiments; bar, SD. (C), Cellular localization of acetylated microtubules (green) in cells exposed to paclitaxel for 6 and 24 h and untreated cells. The cells were stained with acetylated α-tubulin antibody, microtubules (red) with α-tubulin antibody and nuclei (blue) with DAPI. Bar, 50 μm. (D), The ratio of the expression of acetylated α-tubulin to that of α-tubulin obtained with immunofluorescent staining was calculated from integrating each fluorescent intensity on a series of stacked images with a thickness of 1 μm. Columns, mean of value obtained from 3 microscopic fields; bar, SD; ^**^p<0.01 relative to respective untreated (0 h) cells.

Post-translational acetylation of α-tubulin is reported to be associated with the stability of microtubules (21,22). Thus, we evaluated the expression level of acetylated α-tubulin employing the Western blot method (Fig. 6A) and immunocytochemistry (Fig. 6C). The quantification of acetylated α-tubulin and α-tubulin was performed by the same method employed in the tubulin polymerization assay (Fig. 6B). Images obtained from immunofluorescence staining were also analyzed by measuring the fluorescent intensity at each 1-μm deep interval (Fig. 6D). The ratio of the acetylated α-tubulin to the total α-tubulin remained low for 48 h in the II18 cell line, but it increased with time in the RERF-LC-KJ cell line. These analytic results for acetylated α-tubulin were consistent with the results obtained in the tubulin polymerization assay.

### No paclitaxel binding site mutations in the β-tubulin isotype were observed in the 3 cell lines

In order to examine whether alterations of the tubulin gene could account for the different levels of drug sensitivity (resistance) to paclitaxel, we sequenced cDNA from the β-tubulin isotype from the three cell lines. However, no mutations were shown at the paclitaxel binding sites of the β-tubulin isotypes in any of the cell lines examined (data not shown).

### Differences in the intracellular localization of paclitaxel are observed between RERF-LC-KJ and the other cell lines

**Figure 7:**
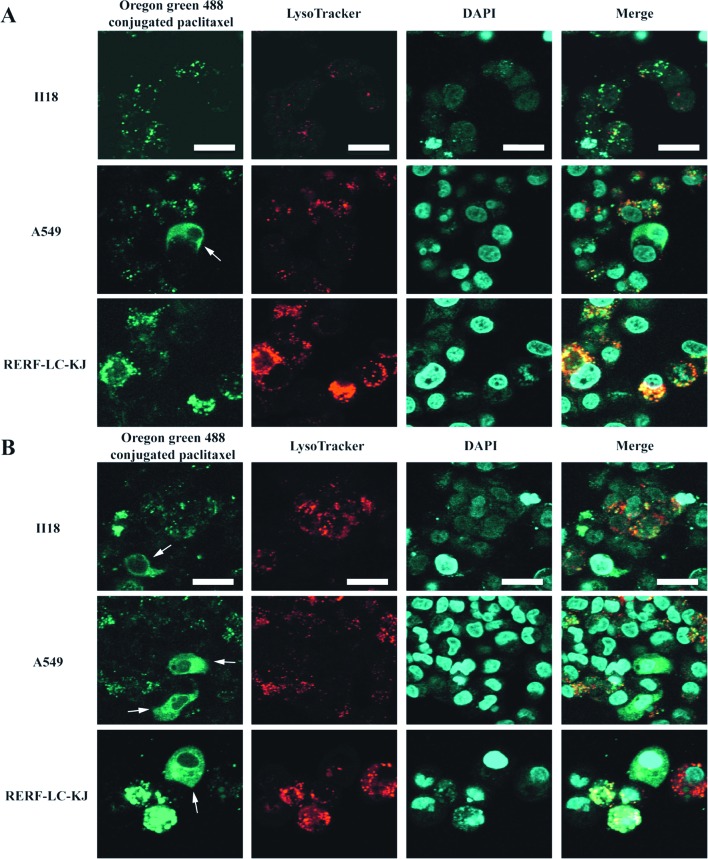
Oregon Green^®^ 488-conjugated paclitaxel made it possible to visualize not only the normal microtubules formation, but also the aggregated vesicle formation in some of the RERF-LC-KJ cells, whereas in the other cell lines these vesicular formations were not remarkable. (A and B), Representative confocal images of lung cancer cell lines after the incubation of Oregon Green^®^ 488 conjugated paclitaxel for 6 and 24 h, respectively, followed by staining with 100 nM LysoTracker^®^ DND-99 (red) and DAPI (blue). Cellular microtubules are highlighted by white arrows. Bars, 20 μm.

In order to study the dynamics of the paclitaxel binding to β-tubulin and microtubules, we performed live cell imaging using Oregon Green^®^ 488 conjugated paclitaxel in living cells. As shown in Fig. 7, Oregon Green^®^ 488 conjugated paclitaxel made it possible to visualize not only the normal microtubule formation on the partial cells, but also the aggregated vesicle formation in some of the RERF-LC-KJ cells, whereas in the other cell lines this phenomenon was not remarkable. Because these vesicular structures did not appear to be composed of microtubules, we conducted tests to determine what cell organelles were associated with these structures, by employing Oregon Green^®^ 488 conjugated paclitaxel and LysoTracker^®^ Red. It turned out that colocalization between Oregon Green^®^ 488 conjugated paclitaxel with LysoTracker^®^ Red was more frequent in the RERF-LC-KJ cells, compared with the other cell lines. Moreover, in the RERF-LC-KJ cells, Oregon Green^®^ 488 conjugated paclitaxel accumulated more in the intracellular compartments, except the lysosomes, compared with the other cell lines.

### The pharmacokinetics profile for paclitaxel varies between the cell lines

We summarized the characteristics of the pharmacokinetics for paclitaxel in each cell line from four viewpoints, including the accumulation of paclitaxel, the extent of *ABCB1* expression, the quantity of the microtubules to which paclitaxel binds, and the volume of paclitaxel which accumulates in the intracellular compartments instead of binding to microtubules. As shown in Table III, A549 exhibited a higher expression level of *ABCB1*, which resulted in less accumulation of paclitaxel, compared with the other cells. In the RERF-LC-KJ cell line, the expression of *ABCB1* was low, and the amount of paclitaxel accumulated intracellularly increased with time. In addition, the rate of tubulin polymerization was maintained at a stable level after the exposure to paclitaxel. The paclitaxel that did not bind to tubulin accumulated preferentially in the intracellular compartments containing lysosomes, in the RERF-LC-KJ cell line. The II18 cell line, the most sensitive to paclitaxel of the cell lines so far examined, showed unique characteristics, since it exhibited lower levels of expression of *ABCB1*, lower accumulation of paclitaxel, little polymerization of tubulin due to paclitaxel, and little accumulation of paclitaxel in the intracellular compartments.

## Discussion

We demonstrated that a remarkable difference existed in the IC_50_ values for paclitaxel among the cell lines derived from human non-small cell lung cancers, by using two cytotoxicity assays. Among the cell lines we examined, RERF-LC-KJ exhibited the highest resistance to paclitaxel.

In order to study the underlying mechanism of such high resistance, as seen in RERF-LC-KJ, we studied the time course of the paclitaxel accumulation and the ratio of polymerized tubulin to total tubulin. We found that the cells showed a continuous uptake of paclitaxel with time and an increase in the ratio of polymerized tubulin, with a peak value at 12 h after the exposure, followed by a gradual decline until 48 h. No mutations were demonstrated in the gene coding for the paclitaxel binding site of the β-tubulin isotypes. Quantitative RT-PCR did not show any overexpression of *ABCB1* or *ABCC1*. It is noteworthy that the aggregated vesicular structures containing paclitaxel colocalized with lysosomes. Thus, these results might suggest that cells resistant to paclitaxel possess some vesicular compartments including lysosomes for sequestrating paclitaxel, thereby hindering paclitaxel from binding to β-tubulin.

Lysosomes, one of the intracellular acidic organelles, are known to play critical roles in producing resistance to anticancer drugs, since they trap drugs with weak basic properties in normal cells (23–27), whereas in several cancer cells, the acidification mechanisms of lysosomes were defective, and so the capacity to sequester lysosomotrophic agents was diminished (26). As a result of the low pH gradient between cytosol and lysosomes, cancer cells have a greater tendency to accumulate drugs in the extra-lysosomal compartments of cells. Paclitaxel, however, is a neutral molecule, because it has no structure to be ionized. Therefore, the pH gradient between the lysosomes and cytosol does not seem to be associated with the intracellular dynamics of paclitaxel. The nature of the extra-lysosomal compartments accumulating paclitaxel currently remains unknown, but it is tempting to speculate that the structures may possess some drug transporters involved in the efflux or uptake of paclitaxel.

The A549 cell line maintained a higher expression of *ABCB1* through 48 h, compared with the other cell lines. Thus, paclitaxel accumulation remained lower in A549, compared with the other cell lines. When we applied the compartment model to the A549 cell line, the cells showed few compartments, so paclitaxel could effectively bind to β-tubulin. These results were compatible with those seen in reports by Alvarez *et al* (28), Ambudkar *et al* (3) and Huang and Sadee (4). On the other hand, it is speculated that there is little association between the expression of *ABCC1* and the drug resistance to paclitaxel.

The II18 cell line was the most sensitive to paclitaxel, among the cell lines so far examined. Nevertheless, it accumulated a considerable amount of paclitaxel, with a peak at 18 h after the exposure and tubulin polymerization proceeded little even at 48 h after the exposure. We thus conclude that the II18 cell line has a unique feature in terms of its sensitivity to paclitaxel. It would be worth conducting further studies on the intracellular localization of paclitaxel in II18 cells, because it might lead to the identification of a novel molecular target for therapy.

In conclusion, there are multiple underlying mechanisms involved in the development of resistance to paclitaxel, and the predominant factors vary between cell lines. In addition to the mechanisms already proven to be involved with drug resistance, which include drug efflux transporters and mutations of the paclitaxel binding site of β-tubulin, we hereby propose that a novel mechanism also exists, in which paclitaxel accumulates in some intracellular compartments, which could play an important role in producing resistance to paclitaxel.

## Figures and Tables

**Table I:** Set of primers for PCR amplification and nucleotide sequencing of β-tubulin isotype.

Primer	Sequence for PCR amplification	Primer	Sequence for sequencing reaction
Class I			
Forward (88–108)	ATACATACCTTGAGGCGAGCA	Forward (687–706)	CTCTCCGTCCATCAGTTGGT
Reverse (1488–1507)	GACGGCTAAGGGAACTGAGA	Reverse (1017–1036)	ACAGGCAGCCATCATGTTCT
Class II			
Forward (83–100)	CACCATGCGCGAGATCGT	Forward (702–721)	GCCCTGTATGACATCTGCTT
Reverse (1461–1480)	CCATGCTTGAGGACAACAGA	Reverse (1052–1070)	CTCGTCCACCTCCTTCATG
Class III			
Forward (65–84)	TATGAGGGAGATCGTGCACA	Forward (644–661)	GGAGAACACGGATGAGAC
Reverse (1462–1482)	GGTTTAGACACTGCTGGCTTC	Reverse (944–962)	CATGTTCTTGGCATCGAAC
Class IVa			
Forward (22–39)	TCTCCGCCGCATCTTCCA	Forward (683–703)	GGTGGAGAATACGGATGAGAC
Reverse (1472–1492)	ATCAAAGGTCAGAAGCCTCGA	Reverse (983–1004)	CATGTTCTTGGCATCGAACATC
Class IVb			
Forward (50–71)	TCTGCTGCTGTTTGTCTACTTC	Forward (636–654)	AGTGGTGGAGCCCTACAAC
Reverse (1461–1480)	GTTCACACTGCTTCCCTGCT	Reverse (985–1005)	AGCCATCATGTTCTTGGCATC

**Table II:** Cytotoxicity profile of lung cancer cell lines to paclitaxel.

		IC_50_^a^						
	
Exposure time (h)		II18	RERF-LC-Ad1	PC-14	A549	RERF-LC-Ad2	Lu99B	RERF-LC-KJ
	24	−8.32	−8.10	−7.67	−7.73	−8.29	−5.60	−3.70
xCELLigence^®^	48	−8.33	−7.78	−7.65	−7.69	−8.15	−5.10	−4.51
	72	−8.18	−8.41	−7.69	−7.07	−7.99	−5.35	−4.83
WST-8^b^	48	−10.80	−9.36	−9.34	−8.48	−7.26	−7.68	−4.48

a IC_50_, drug concentration (in logM) at which cell growth is inhibited by 50%;

b WST-8, 2-(2-methoxy-4-nitrophenyl)-3-(4-nitrophenyl)-5-(2,4-disulfophenyl)-2H-tetrazolium.

**Table III:**
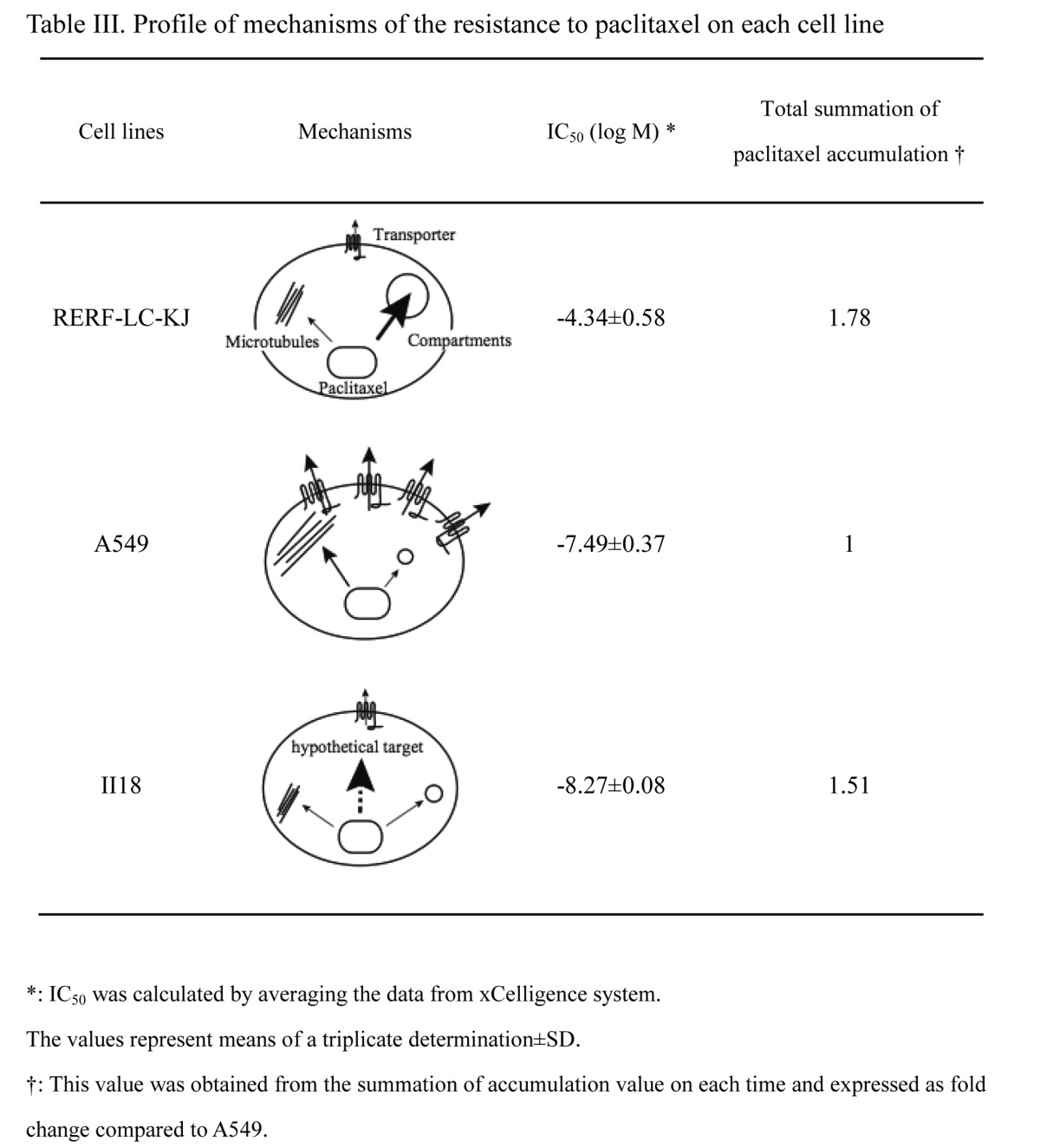
Profile of mechanisms of the resistance to paclitaxel on each cell line.
